# Assessing the Risk of Type 2 Diabetes Among University Employees in Kuwait: A Cross-Sectional Study

**DOI:** 10.3390/ijerph23040455

**Published:** 2026-04-02

**Authors:** Saleh Alsarhan, Fatema Alkhulaifi, Mai Alhazami, Mashael M. Alshammari, Fatemah M. Alotaibi, Nouf A. Alajmi, Anfal F. Almutairi, Joud H. Almutairat

**Affiliations:** 1 Department of Health Policy and Management, College of Public Health, Kuwait University, Kuwait City 13060, Kuwait; 2 Department of Epidemiology and Biostatistics, College of Public Health, Kuwait University, Kuwait City 13060, Kuwait; fatemah.alkhulaifi@ku.edu.kw; 3 Department of Pharmacy Practice, College of Pharmacy, Kuwait University, Kuwait City 13060, Kuwait; mai.alhazami@ku.edu.kw; 4 Department of Public Health Practice, College of Public Health, Kuwait University, Kuwait City 13060, Kuwait; s2211119095@ku.edu.kw (M.M.A.); s2211120596@ku.edu.kw (F.M.A.); s2211123201@ku.edu.kw (N.A.A.); s2211116939@ku.edu.kw (A.F.A.); s2211138623@ku.edu.kw (J.H.A.)

**Keywords:** type 2 diabetes mellitus, FINDRISC, risk score, screening

## Abstract

**Highlights:**

**Public health relevance—How does this work relate to a public health issue?**

- This study addresses the high burden of type 2 diabetes in Kuwait by assessing diabetes risk among university employees using a validated, non-invasive screening tool.

**Public health significance—Why is this work of significance to public health?**

- Approximately one-third of university employees were classified as being at an increased 10-year risk of developing type 2 diabetes, indicating a substantial hidden burden even within a highly educated workforce.

**Public health implications—What are the key implications or messages for practitioners, policy makers and/or researchers in public health?**

- Workplace-based diabetes risk assessment can support early identification of employees classified as being at higher risk and inform targeted prevention strategies and workplace health policies in Kuwait and similar high-risk settings.

**Abstract:**

Type 2 diabetes mellitus (T2DM) is a major global public health concern, with Kuwait experiencing one of the highest prevalence rates worldwide, affecting approximately one quarter of adults. The Finnish Diabetes Risk Score (FINDRISC) is a simple, cost-effective screening tool for identifying individuals at high risk of developing T2DM. This study aimed to assess the risk of T2DM among Kuwait University (KU) employees using the FINDRISC and to identify associated predictors. A cross-sectional study was conducted on 407 KU employees. Data were collected in person, including anthropometric measurements. Participants with a FINDRISC score ≥ 12 were classified as being at increased risk of T2DM. Multivariable logistic regression was used to identify predictors of increased risk. Findings revealed a mean FINDRISC score of 9.4, with females having a significantly higher score than males (10.3 and 8.2, respectively), indicating a higher risk of T2DM. Overall, 137 participants (33.7%) were classified as being at increased 10-year risk of developing T2DM. Increased risk was significantly associated with being female (aOR: 2.85, 95% CI: 1.55–5.24), being married (aOR: 2.92, 95% CI: 1.57–5.43), and having higher perceived susceptibility to diabetes, including very likely (aOR: 7.73, 95% CI: 3.32–18.02) or somewhat likely (aOR: 2.41, 95% CI: 1.35–4.32) compared with not at all likely. Overall, approximately one-third of KU employees were classified as being at an increased risk of T2DM. These findings suggest the potential value of workplace-based screening and diabetes prevention programs to promote early detection and facilitate the early identification of individuals who may benefit from preventive interventions.

## 1. Introduction

Diabetes is a chronic condition that occurs when the body cannot produce enough insulin to regulate blood glucose levels [1]. In 2021, diabetes was responsible for 1.6 million deaths globally [2]. Furthermore, diabetes has been determined to be the cause of 0.5 million kidney disease deaths and 11% of cardiovascular disease deaths [2]. In 2024, around 500 million adults worldwide were living with diabetes [3]. Kuwait is among the countries with the highest burden, with an age-standardized prevalence of 25.6% among adults aged 20–79 years, ranking third globally [3].

Diabetes may remain asymptomatic for years before clinical diagnosis. A joint World Health Organization (WHO) and International Diabetes Federation (IDF) report on screening for type 2 diabetes highlighted the prolonged asymptomatic phase of the disease and discussed the potential advantages of targeting high-risk individuals when considering screening strategies [4]. In the Middle East and North Africa (MENA) region, approximately 37% of adults are unaware that they have diabetes [3]. Early detection of diabetes is therefore essential, as early control and intervention are associated with improved health outcomes [5]. Furthermore, early detection of the disease is associated with a lower economic burden, as a study in the US identified diabetes as one of the largest contributors to healthcare costs [6]. Multiple factors have been associated with an increased risk of developing diabetes. These factors include age, obesity, gender, marital status, and economic status [7]. By utilizing some of these factors, several non-invasive assessment methods have been established to aid in the screening and risk assessment of diabetes, including the Finnish Diabetes Risk Score (FINDRISC) [8].

Several studies across different populations in the region have applied the FINDRISC to estimate the 10-year risk of developing type 2 diabetes mellitus (T2DM) [9,10,11,12,13,14,15,16,17,18,19,20,21]. In Kuwait, diabetes risk scores, including FINDRISC-based approaches, have been evaluated against biochemical testing and demonstrated acceptable diagnostic performance for identifying undiagnosed diabetes, with approximately 4% of adults living with undiagnosed diabetes and 20% having impaired blood glucose [14]. Awad et al. (2015) reported that approximately 30% of participants were classified as having a moderate to high 10-year risk of developing T2DM [15]. Furthermore, a workplace-based study among hospital employees demonstrated that FINDRISC-guided screening detected previously undiagnosed diabetes (11.8%) and prediabetes (51%) among high-risk employees [16]. These findings highlight the utility of FINDRISC as a practical and scalable screening tool in regional and workplace settings.

While prior studies have investigated the 10-year risk of T2DM in the general population and selected occupational groups in Kuwait, these investigations primarily provide population-level estimates but offer limited insight into risk distribution within a defined institutional workforce. University employees represent a distinct and understudied professional population characterized by heterogeneous academic and administrative roles, variable occupational physical activity, and potentially prolonged sedentary work patterns that may shape cardiometabolic risk differently from previously examined groups. Institution-specific assessments are particularly relevant in academic settings, where structured organizational environments allow for the integration of targeted screening and prevention initiatives.

To date, no study has assessed the 10-year risk of developing T2DM among Kuwait University (KU) employees. Generating updated, workforce-specific risk stratification data extends prior national findings by identifying the magnitude and determinants of risk within a large academic institution, thereby providing actionable evidence for context-tailored prevention planning. In addition, limited research has examined how perceived susceptibility to diabetes and perceptions of workplace health and wellbeing support relate to objectively estimated risk in occupational settings, despite their relevance for designing effective prevention and risk communication strategies.

Accordingly, the present study aimed to assess the 10-year risk of developing T2DM among KU employees using the FINDRISC tool and to identify sociodemographic, occupational, and perception-based predictors associated with increased diabetes risk.

## 2. Materials and Methods

Study Design, Settings, and Participants: This cross-sectional study was conducted at KU using a structured questionnaire adapted from the FINDRISC tool, which includes items on age, body mass index (BMI), waist circumference, physical activity, diet, hypertension medication, blood sugar history, and family history of diabetes. A pilot test was conducted to ensure clarity.

The sample size was calculated, given that there are approximately 6870 employees at KU, with a 5% margin of error and assuming a 30% prevalence of diabetes risk from a prior study in Kuwait [15]. The estimated required sample size was 308 using a single proportion formula. Data collection was carried out from February to April 2025, and a total of 407 participants were included in the final analysis. Both English and Arabic versions of the questionnaire were available to optimize comprehension for the participants (Supplementary Table S1). Ethical approval for the study was obtained from the Health Sciences Center (HSC) Ethical Committee for Student Research Projects at KU on 18 December 2024 (Ref: 831).

Participants were recruited through convenience sampling using a structured, in-person outreach strategy across all KU campuses. An official letter was sent to the deans of all KU colleges to facilitate the investigators’ ability to conduct on-site data collection within academic and administrative departments. Following institutional approval, investigators rotated across campuses and departments during standard working hours. Recruitment locations and times were distributed to minimize overrepresentation of specific units and to reduce potential selection bias related to site and occupational type.

Inclusion criteria were current employment at KU and provision of informed consent. Exclusion criteria included being pregnant or a prior diagnosis of diabetes because FINDRISC is designed to estimate future risk among individuals without established T2DM. Employees were first provided with study information and invited to participate. Individuals who declined participation were not screened further. Those who agreed provided informed consent and subsequently completed brief screening questions to confirm eligibility. The refusal rate was calculated as the proportion of employees who declined participation and refused to consent. Eligible participants then completed the questionnaire. After data collection, responses were reviewed for completeness prior to inclusion in the final analytical sample.

Measures: The primary outcome measure in this study was the FINDRISC score, which ranges from 0 to 26 points, with higher scores indicating greater risk of developing T2DM. Each FINDRISC item includes predefined response categories with assigned score points based on the FINDRISC scoring algorithm. The FINDRISC score was calculated by summing FINDRISC individual item scores, with each component weighted according to its established contribution to diabetes risk. Participants were initially classified using standard FINDRISC categories (low < 7 points, slightly elevated 7–11 points, moderate 12–14 points, high 15–20 points, or very high > 20 points) [8,15].

For analytical purposes, FINDRISC scores were dichotomized into not increased risk (≤11 points) and increased risk (≥12 points), corresponding to the points threshold defining the lower boundary of the moderate-risk category in the original FINDRISC classification. This classification aligns with prior studies [22,23,24,25,26], including research conducted in Kuwait [15], and facilitates direct comparison with existing literature. Contemporary longitudinal evidence further supports use of this threshold to capture the majority of future diabetes cases [27]. Throughout the manuscript, the term “increased risk” refers to participants classified within the moderate-to-very high FINDRISC categories. Dichotomization was used to facilitate group comparisons and enhance interpretability within a workplace screening context (Supplementary Table S2).

In Kuwait, a population-based study by Al-Khalaf et al. evaluated FINDRISC against biochemical screening and reported lower FINDRISC thresholds (≥9 points) were associated with higher sensitivity for detecting undiagnosed diabetes, underscoring the importance of context-specific cut-off selection [14]. Similarly, recent meta-analytic evidence suggests that thresholds around 9–10 points optimize screening sensitivity, whereas higher thresholds (≥12 points) improve specificity and reduce false-positive classifications [28]. Given the structured nature of workplace screening and the need to efficiently identify individuals at greater estimated risk, the ≥12 threshold was selected as the primary classification in this study. A sensitivity analysis was conducted using the lower threshold (≥9 points) to evaluate the robustness of the observed associations (Supplementary Table S3).

In addition to the FINDRISC items, the study incorporated a range of sociodemographic, occupational, and behavioral characteristics, including gender, nationality, marital status, education level, professional position, and smoking status to examine predictors of increased diabetes risk. Smoking status was categorized as never smoker, former smoker, and current smoker in accordance with Centers for Disease Control and Prevention definitions [29]. The anthropometric measures included height, weight, and waist circumference. These measurements were taken by the research team to ensure accuracy. Waist circumference categories were operationalized using sex-specific thresholds as defined in the FINDRISC scoring algorithm (men: <94, 94–102, >102 cm; women: <80, 80–88, >88 cm). These thresholds correspond to the Europid cut-points (≥94 cm for men and ≥80 cm for women) recommended by the IDF for Middle Eastern populations pending region-specific data [30]. BMI was calculated using the standard formula: weight (kg) divided by height in meters squared (m^2^). Two perception-based questions were included: (1) participants rated how likely they are to develop diabetes in the next ten years, considering their lifestyle factors and their family medical history; and (2) participants assessed the extent to which they believe their workplace supports employees in overcoming barriers related to health and wellbeing.

Statistical Analysis: Descriptive statistics were used to summarize participant characteristics. Categorical variables were reported as frequencies and percentages, while the continuous variable (FINDRISC score) was summarized using means and standard deviations (SDs). Independent samples *t*-tests were used to compare mean FINDRISC scores between groups including comparisons by gender. Chi-square tests were used to examine associations between categorical variables. Because these comparisons were limited in number, pre-specified, and intended to provide descriptive and exploratory comparisons by gender rather than to test a single primary hypothesis, adjustment for multiple testing was not applied.

The primary inferential analyses were based on regression models. Multivariable logistic regression analysis was used to identify independent predictors of increased risk of developing T2DM, with results reported as adjusted odds ratios (aORs) with 95% confidence intervals (CI). Individual FINDRISC components (e.g., age, BMI, waist circumference, family history of diabetes) were not included in the regression modeling to avoid circularity and mathematical coupling with the composite FINDRISC-derived outcome. Variables were selected a priori based on prior literature, established risk factors for T2DM, and theoretical relevance, rather than being selected solely based on statistical significance in univariate analyses [7,15,31]. Multivariable logistic regression was used to account for potential confounding among sociodemographic and perception-related variables. All key variables were retained in the model regardless of statistical significance to ensure appropriate adjustment. Model diagnostics were performed to assess multicollinearity, model fit, and discriminatory ability. Variance inflation factors (VIF) were examined to assess multicollinearity among independent variables. Model goodness-of-fit was evaluated using the Pearson chi-square test. Discriminatory performance was assessed using the area under the receiver operating characteristic (ROC) curve. A *p*-value < 0.05 was considered statistically significant. All analyses were performed using STATA version 14.2. (StataCorp LLC, College Station, TX, USA).

## 3. Results

**Figure 1:**
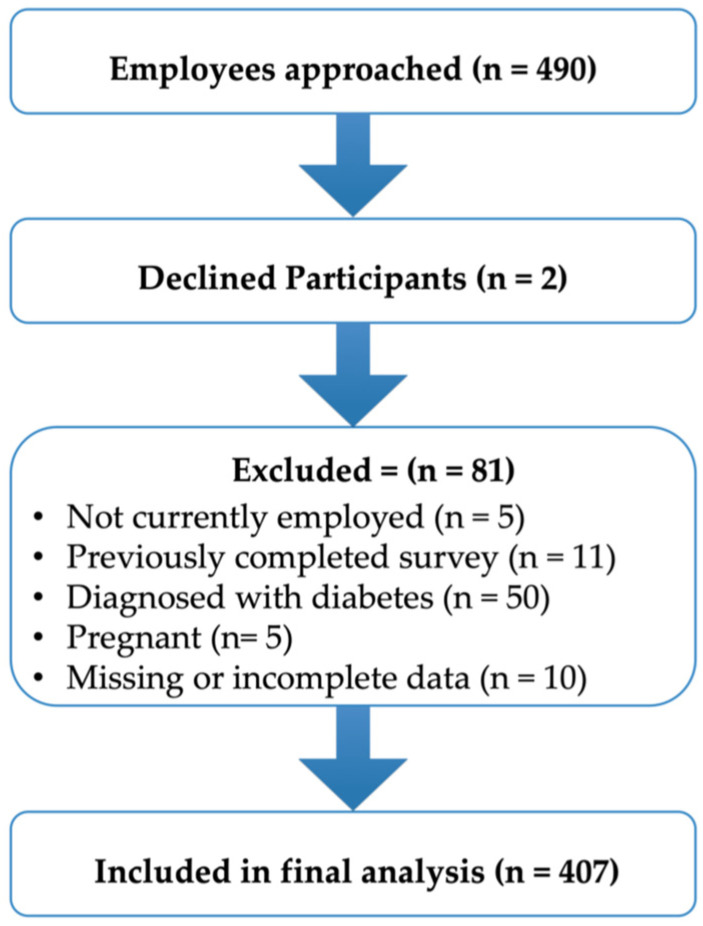
Flow diagram of participant recruitment and inclusion.

A total of 490 employees were initially approached to participate; of those, 83 were excluded due to ineligibility, leaving 407 eligible participants for inclusion in the final analysis as shown in Figure 1. Among employees approached, only two declined participation, resulting in a refusal rate of approximately 0.4%. Table 1 presents the general characteristics of the study population and their distribution by gender. Overall, approximately 58.0% of participants were female, 64.9% were aged 18–44 years, and the majority were Kuwaiti (67.3%). Approximately 84.8% of the participants had never smoked. More than half of the participants were either overweight (38.3%) or obese (20.2%) based on BMI.

Several variables differed significantly between males and females. The distribution of age categories differed significantly by gender (*p* < 0.001). A higher proportion of females than males were in the 18–44-year age group (74.2% vs. 52.1%), whereas males were more represented in the >54-year age group (22.8% vs. 6.4%). In addition, significant gender differences were observed in nationality, marital status, professional position, education level, and smoking status (all *p*-values ≤ 0.049). Females were more often Kuwaiti, single, non-academic, and non-smokers, whereas males were more often non-Kuwaiti, married, held academic roles, highly educated, and more likely to smoke.

Perceived diabetes risk and perception of workplace health and wellbeing support did not differ significantly by gender (*p*-value = 0.544 and *p*-value = 0.426, respectively). Overall, 41.5% of participants perceived themselves as somewhat likely to develop T2DM within the next ten years. The largest proportion of participants expressed neutral views (32.2%) on workplace health and wellbeing support.

Furthermore, several clinical and lifestyle FINDRISC components differed significantly by gender. Waist circumference categories, history of high blood glucose, family history of diabetes, and medication for blood pressure differed significantly by gender (*p*-value < 0.001, *p*-value = 0.004). In contrast, BMI distribution, daily physical activity (≥30 min), and daily fruit and vegetable consumption did not differ by gender (*p*-value = 0.067, *p*-value = 0.052, and *p*-value = 0.414, respectively). When comparing FINDRISC scores by gender, females demonstrated a significantly higher mean FINDRISC score than males (10.3 vs. 8.2, *p*-value < 0.001).

The distribution of FINDRISC categories and dichotomized 10-year risk of T2DM among the study population is presented in Table 2. The largest proportion of participants (38.6%) was in the “slightly elevated risk” category. When the five categories were dichotomized into increased risk (≥12) versus not increased risk (≤11), approximately one-third of the study population (33.7%) was classified as being at increased risk of developing T2DM within the next ten years.

The results of the multivariable logistic regression model for identifying predictors of increased risk of T2DM among the study population are presented in Table 3. Females had 2.85 times the odds of being classified as at increased 10-year T2DM risk compared to males (females vs. males aOR: 2.85, 95% CI: 1.55–5.24). Moreover, marital status was found to be a significant predictor of increased risk with T2DM. Specifically, being married was associated with higher odds of increased risk compared to being single (married vs. single aOR: 2.92, 95% CI: 1.57–5.43). Furthermore, there was a strong association between perception of diabetes risk and being classified at increased risk of developing T2DM. Participants who perceived themselves as very likely to develop diabetes had approximately 8 times greater odds for being classified at increased risk to develop T2DM compared to those who did not hold this belief (very likely vs. not at all likely aOR: 7.73, 95% CI: 3.32–18.02), and participants who reported that they believe they were somewhat likely to develop diabetes had more than twice the odds of being classified in the increased risk group compared to those who did not (somewhat likely vs. not at all likely aOR: 2.41, 95% CI: 1.35–4.32). No significant association was observed between employees’ perception of workplace health and well-being support and classification in the increased T2DM risk group. Model diagnostics indicated no evidence of problematic multicollinearity, with a mean VIF of 2.05 and all individual VIF values below 5. The Pearson goodness-of-fit test suggested adequate model fit (χ^2^ = 261.66, *p* = 0.130). The model demonstrated acceptable discriminatory ability, with an area under the ROC curve of 0.73. Findings from the sensitivity analysis using the ≥9-point threshold were directionally consistent with the primary analysis (Supplementary Table S3); however, nationality was statistically significant suggesting some threshold-dependent variability.

## 4. Discussion

While national estimates of diabetes risk in Kuwait have been previously reported, institutional-level assessments remain limited. Translating population-level data into effective prevention strategies requires workforce-specific risk profiling that reflects the structure and context of defined occupational settings. In this study, approximately one-third (33.7%) of KU employees were classified as being at increased 10-year risk of T2DM, indicating a substantial burden within a large academic workforce. By integrating objective risk stratification with perception-based measures, this study extends prior work beyond prevalence estimation to provide context-specific evidence for targeted occupational health prevention.

Our findings are broadly consistent with studies conducted both within the Middle East and internationally. A population-based study conducted in Kuwait by Awad et al. examined T2DM risk in the general population and reported FINDRISC scores comparable to ours, with 30% of adults identified as being at increased risk for developing T2DM [15]. In Saudi Arabia, a similar study assessed the risk of T2DM in the general population and reported that 18% were at increased risk for developing T2DM [9]. Beyond the MENA region, a university-based study conducted in Turkey showed that more than half of employees were at increased risk of developing T2DM, especially among academic staff [32]. Furthermore, a study in Norway also indicated that about one-third of adults are at increased risk of developing T2DM [33]. Together, these findings reinforce the broad applicability and reliability of the FINDRISC tool across diverse populations and institutional settings, particularly in regions with a high burden of T2DM.

In this study, we identified the main predictors associated with increased risk for developing T2DM among KU employees. Compared to male participants, females had more than threefold higher odds of increased T2DM risk. These results are in agreement with previous research from Kuwait where women had higher T2DM risk than men [15]. This association may reflect differences in adiposity, particularly central obesity, as well as lower levels of physical activity. It is important to note that the FINDRISC scoring algorithm incorporates sex-specific waist circumference cutoffs, with lower thresholds applied for women than for men. As waist circumference contributes directly to the total score, women with comparable levels of central adiposity may accumulate higher FINDRISC scores relative to men. Therefore, part of the observed association between female sex and increased risk classification may reflect structural properties of the scoring algorithm rather than a fully independent biological effect.

Additionally, marital status was associated with FINDRISC-classified risk of developing T2DM, with a higher proportion of married employees classified as at increased risk compared to non-married employees, consistent with findings from a national population study conducted by Awad et al. (2015) [15]. This pattern may reflect lifestyle characteristics commonly observed after marriage, including reduced physical activity, gradual weight gain, and dietary changes, which are associated with cardiometabolic risk. Furthermore, our study revealed an important finding whereby employees’ perceived risk of developing T2DM was strongly associated with higher FINDRISC scores and were almost eight times more likely to fall into the increased risk category of developing T2DM within the next ten years. The strong association between perceived susceptibility to diabetes and FINDRISC classification may reflect participants’ awareness of existing cardiometabolic risk factors that are directly captured within the FINDRISC scoring algorithm, such as age, adiposity, and family history. Thus, perceived risk may represent alignment between subjective appraisal and objective risk estimation rather than an independent causal predictor. In addition, because perceived risk was self-reported, the observed association may partly reflect response bias, whereby individuals who are aware of their underlying risk factors report higher perceived susceptibility. This interpretation is consistent with findings from Hivert et al., who demonstrated that individuals with higher perceived diabetes risk were at objectively higher metabolic risk; however, higher perceived risk did not translate into greater intention to adopt healthier lifestyle behaviors, suggesting that perceived susceptibility to diabetes alone may be insufficient to drive behavioral change [34]. These findings suggest that both sociodemographic characteristics and perceived susceptibility to diabetes are associated with higher risk classification and may be relevant when designing targeted workplace prevention strategies.

The substantial proportion of employees classified at increased risk of T2DM highlights the potential relevance of workplace-based prevention strategies. Consistent with screening recommendations [35], risk-based tools such as FINDRISC offer a pragmatic approach to identifying individuals who may benefit most from preventive interventions while avoiding unnecessary biochemical testing in low-risk groups. Although screening alone has limited evidence for reducing diabetes-related morbidity, its primary value lies in facilitating early, targeted prevention rather than diagnosis. Occupational evidence supports the feasibility of this approach; Gyberg et al. demonstrated that incorporating FINDRISC into an online workplace survey achieved high participation rates and effectively identified employees at elevated risk for follow-up intervention [36]. Universities represent particularly suitable environments for such initiatives due to their structured workforce and institutional health infrastructure. Structured workplace lifestyle programs have been shown to improve diabetes-related risk factors [37]. In the Finnish Diabetes Prevention Study, intensive lifestyle intervention reduced diabetes by 58% over 3.2 years [38]. Similarly, a large occupational study in France found that combining FINDRISC-based screening with structured telephone coaching was feasible and associated with improvements in dietary behaviors and quality of life among high-risk employees [39]. In alignment with these findings, the World Health Organization emphasizes that maintaining healthy body weight, engaging in regular physical activity, and adopting a balanced diet are effective strategies for preventing or delaying T2DM [1]. Integrating periodic risk assessment with structured lifestyle support within academic settings may represent a practical and scalable prevention strategy.

The observed risk distribution highlights an opportunity for targeted, risk-stratified prevention within the university setting. Rather than broad educational campaigns, institutional efforts could prioritize employees classified at increased risk through structured initiatives such as on-campus risk reassessment campaigns, facilitated referral pathways to primary care, personalized lifestyle counseling, and workplace-based physical activity or nutrition programs. Integrating FINDRISC screening within existing employee health services may allow institutions to allocate preventive resources more efficiently while focusing support on those most likely to benefit. In high-prevalence contexts such as Kuwait, such institution-based approaches may contribute meaningfully to early diabetes prevention efforts.

This study has several strengths. It focuses on an under-researched occupational population in Kuwait and was conducted at KU, the largest academic institution in the country that employs approximately 6870 individuals and enrolls around 44,000 students. The relatively large sample size and low refusal rate may reduce, but do not eliminate, the risk of selection bias. Additionally, this study is among the first in Kuwait and the region to examine the association between perceived diabetes risk and FINDRISC-based risk classification. The use of FINDRISC, a validated and widely applied non-invasive screening tool across diverse populations, including Middle Eastern settings [8,10,12,13,16,17,18,19,20,21,22,23,24,32,39,40,41,42], further strengthens the study. In Kuwait, a population-based screening study by Al-Khalaf et al. evaluated FINDRISC against biochemical testing and reported a sensitivity of 77% and specificity of 66% for detecting undiagnosed diabetes. Similarly, a university-based validation study conducted in Lebanon compared FINDRISC with fasting blood glucose and oral glucose tolerance testing and demonstrated a sensitivity of 83.3% and specificity of 61.3% at the optimal threshold for identifying undiagnosed type 2 diabetes [13]. These regional findings collectively support the acceptable diagnostic performance and practical utility of FINDRISC for diabetes risk stratification in Middle Eastern workplace populations.

This study has several limitations. First, its cross-sectional design precludes causal inference; therefore, observed associations should be interpreted as correlates rather than determinants. Although multivariable regression was used to adjust for key sociodemographic variables, residual confounding from unmeasured or imprecisely measured factors (e.g., dietary patterns, physical activity intensity, or genetic predisposition) may persist. Second, given that participants were recruited from a single academic institution using convenience sampling, findings should be interpreted with possible selection bias and a limited generalizability beyond KU. Since this study did not use probability sampling, there is a possibility of overrepresentation of certain employees, even with approvals and recruitment from all campuses. The relatively high proportion of highly educated participants further restricts extrapolation to the broader working population in Kuwait. Accordingly, findings should be interpreted as institution-specific risk estimates rather than population-level prevalence. Third, the outcome was derived from the FINDRISC tool and was not confirmed using biochemical markers such as fasting glucose or HbA1c, which may introduce misclassification of participants’ risk status. While FINDRISC has demonstrated acceptable validity in regional populations, it has not been specifically calibrated among university employees in Kuwait. Importantly, FINDRISC is a validated screening tool designed to identify individuals at increased future risk of T2DM rather than to establish a clinical diagnosis. Therefore, the findings reflect risk stratification rather than confirmed disease status, and individuals classified as high risk may benefit from follow-up assessment using standard biochemical testing. Fourth, FINDRISC components (e.g., age, BMI, waist circumference, and family history of diabetes) were not included in the regression models because they are directly incorporated into the composite outcome. Excluding these variables was necessary to avoid circularity and mathematical coupling; however, this approach limits the ability of the model to independently assess the association between these clinically important factors and the outcome. Finally, some variables were self-reported and may be subject to reporting bias. Despite these limitations, the study provides valuable insight into diabetes risk distribution within a defined occupational setting.

Recommendations: Additional studies are needed to evaluate FINDRISC’s predictive performance against blood-based biomarkers in workplace screening programs, particularly in Kuwait and the other GCC countries where population-specific risk profiles may influence performance. Studies assessing the cost-effectiveness of implementing FINDRISC as a routine, non-invasive screening tool are also warranted. Future research should examine the effectiveness of post-screening, workplace-based preventive interventions and linkage to primary care for early prevention of T2DM. Mixed-methods studies incorporating qualitative interviews across FINDRISC risk categories could provide contextual insights into risk perception, workplace influences, and barriers to lifestyle modification, complementing quantitative risk stratification.

## 5. Conclusions

A substantial proportion of KU employees were classified as being at an increased 10-year risk of T2DM. Female sex, marital status, and higher perceived susceptibility were independently associated with increased risk classification. These findings indicate a meaningful burden of future diabetes risk within a large academic workforce and support the role of structured, workplace-based risk assessment and prevention strategies. Embedding risk-stratified approaches within institutional health frameworks may contribute to earlier identification and targeted prevention in high-prevalence settings such as Kuwait.

## Figures and Tables

**Table 1:** General characteristics of the study participants by gender (*n* = 407).

Variable	*n* (%)	Gender		*p*-Value
		Male (%)	Female (%)	
		(*n* = 171)	(*n* = 236)	
Age (years) ^‡^				
18–44	264 (64.9)	89 (52.1)	175 (74.2)	<0.001 *^,^^
45–54	89 (21.9)	43 (25.2)	46 (19.5)	
>54 ^†^	54 (13.3)	39 (22.8)	15 (6.4)	
Nationality				
Kuwaiti	274 (67.3)	82 (48.0)	192 (81.4)	<0.001 *^,^^
Non-Kuwaiti	133 (32.7)	89 (52.1)	44 (18.6)	
Marital status				
Single	102 (25.1)	36 (21.1)	66 (28.0)	0.049 *^,^^
Married	279 (68.6)	128 (74.9)	151 (64.0)	
Divorced/widowed	26 (6.4)	7 (4.1)	19 (8.1)	
Professional position				
Academic	188 (46.2)	104 (60.8)	84 (35.6)	<0.001 *^,^^
Non-academic	219 (53.8)	67 (39.2)	152 (64.4)	
Education level				
2-Year college diploma or less	58 (14.3)	35 (20.5)	23 (9.8)	<0.001 *^,^^
Bachelor’s degree	142 (34.9)	29 (17.0)	113 (47.9)	
Master’s degree	68 (16.7)	17 (9.9)	51 (21.6)	
Doctoral degree and/or professional degree (PhD, MD, DDS, etc.)	139 (34.2)	90 (52.6)	49 (20.8)	
Smoking status				
Never smoked	345 (84.8)	118 (69.0)	227 (96.2)	<0.001 *^,^^
Smoker/Former smoker	62 (15.2)	53 (31.0)	9 (3.8)	
Employee perception of diabetes risk				
Not at all likely	116 (28.5)	53 (31.0)	63 (26.7)	0.544 ^
Somewhat likely	169 (41.5)	69 (40.4)	100 (42.4)	
Very likely	42 (10.3)	14 (8.2)	28 (11.9)	
I don’t know	80 (19.7)	35 (20.5)	45 (19.1)	
Employee perception of workplace health and wellbeing support				
Strongly agree	54 (13.3)	29 (17.0)	25 (10.6)	0.426 ^
Agree	99 (24.3)	38 (22.2)	61 (25.9)	
Neutral	131 (32.2)	52 (30.4)	79 (33.5)	
Disagree	52 (12.8)	22 (12.9)	30 (12.7)	
Strongly disagree	71 (17.4)	30 (17.5)	41 (17.4)	
BMI (kg/m^2^) ^‡^				
<25 ^††^	169 (41.5)	60 (35.1)	109 (46.2)	0.067 ^
25–29.9 (overweight)	156 (38.3)	75 (43.9)	81 (34.3)	
≥30 (obesity)	82 (20.2)	36 (21.1)	46 (19.5)	
Waist circumference (cm) ^‡^				
M < 94; W < 80	183 (45.0)	113 (66.1)	70 (29.7)	<0.001 *^,^^
M 94–102; W 80–88	128 (31.5)	32 (18.7)	96 (40.7)	
M > 102; W > 88	96 (23.6)	26 (15.2)	70 (29.7)	
Daily physical activity, 30 min or more ^‡^				
No	136 (33.4)	48 (28.1)	88 (37.3)	0.052 ^
Yes	271 (66.6)	123 (71.9)	148 (62.7)	
Daily vegetable, fruit, or berry consumption ^‡^				
No	157 (38.6)	62 (36.3)	95 (40.3)	0.414 ^
Yes	250 (61.4)	109 (63.7)	141 (59.8)	
History of high blood glucose ^‡^				
No	326 (80.1)	150 (87.7)	176 (74.6)	<0.001 *^,^^
Yes	81 (19.9)	21 (12.3)	60 (25.4)	
Family history of diabetes ^‡^				
No	91 (22.4)	58 (33.9)	33 (14.0)	<0.001 *^,^^
First relative	213 (52.3)	75 (43.9)	138 (58.5)	
Second relative	103 (25.3)	38 (22.2)	65 (27.5)	
Medication for BP ^‡^				
No	356 (87.5)	140 (81.9)	216 (91.5)	0.004 *^,^^
Yes	51 (12.5)	31 (18.1)	20 (8.5)	
FINDRISC score (mean, SD)	9.4 (4.9)	8.2 (5.1)	10.3 (4.5)	<0.001 *^,+^

*n*: Number of participants; PhD: Doctor of Philosophy; MD: Doctor of Medicine; DDS: Doctor of Dental Surgery; BMI: Body mass index; M: Men; W: Women; SD: Standard deviation; ^: Chi-square test; *: Statistically significant at *p* < 0.05; +: *t*-test; FINDRISC: Finnish Diabetes Risk Score; BP: Blood pressure; ^†^: Seven participants were aged >64 years were grouped with the >54 years category but they were assigned a FINDRISC age score of 4 points for statistical analysis; ^††^: Three participants were underweight (BMI < 18.5) and were grouped with <25 weight category for statistical analysis.; ^‡^: indicates a FINDRISC item which consists of predefined response categories with assigned score points according to the original FINDRISC scoring algorithm; the total FINDRISC score represents the sum of all item points.

**Table 2:** Distribution of FINDRISC categories and dichotomized 10-year risk of T2DM among study participants (*n* = 407).

Dichotomized FINDRISC Group	Employee Risk Score	Estimated Probability of Developing T2DM	*n* (%)
Not increased risk	Low risk (<7)	1 in 100	113 (27.8)
	Slightly elevated risk (7–11)	1 in 25	157 (38.6)
	**Total (≤11)**		**270 (66.3)**
Increased risk	Moderate risk (12–14)	1 in 6	73 (17.9)
	High risk (15–20)	1 in 3	59 (14.5)
	Very high risk (>20)	1 in 2	5 (1.2)
	**Total (≥12)**		**137 (33.7)**

*n*: Number of participants; T2DM: Type 2 Diabetes Mellitus; FINDRISC: Finnish Diabetes Risk Score. Note: Bold values indicate subtotal rows for each dichotomized FINDRISC group.

**Table 3:** Multivariable logistic regression analysis of predictors of increased risk of T2DM among KU employees.

Variable	Adjusted Odds Ratio	95% CI	*p*-Value
**Gender**			
Male	Reference		
Female	2.85	1.55–5.24	0.001 *
**Nationality**			
Kuwaiti	Reference		
Non-Kuwaiti	1.41	0.82–2.42	0.210
**Marital status**			
Single	Reference		
Married	2.92	1.57–5.43	0.001 *
Divorced/widowed	1.92	0.68–5.43	0.218
**Professional position**			
Academic	Reference		
Non-academic	1.52	0.70–3.31	0.291
**Education level**			
2-Year college diploma or less	Reference		
Bachelor’s degree	1.08	0.49–2.39	0.847
Master’s degree	1.60	0.61–4.15	0.337
Doctoral degree and/or professional degree (PhD, MD, DDS, etc.)	2.56	0.88–7.44	0.083
**Smoking status**			
Never smoked	Reference		
Smoker/former smoker	1.07	0.52–2.18	0.857
**Employee perception of diabetes risk**			
Not at all likely	Reference		
Somewhat likely	2.41	1.35–4.32	0.003 *
Very likely	7.73	3.32–18.02	<0.001 *
I don’t know	1.97	0.96–4.05	0.064
**Employee perception of workplace health and wellbeing support**			
Strongly agree	Reference		
Agree	1.02	0.46–2.27	0.961
Neutral	0.65	0.30–1.45	0.296
Disagree	1.52	0.63–3.69	0.354
Strongly disagree	1.09	0.46–2.59	0.843

CI: Confidence interval; PhD: Doctor of Philosophy; MD: Doctor of Medicine; DDS: Doctor of Dental Surgery; *: Statistically significant at *p* < 0.05; T2DM: Type 2 Diabetes Mellitus.

## Data Availability

The dataset used in this study is available in the Supplementary Materials. Further inquiries can be directed to the corresponding author.

## Supplementary Materials

The following supporting information can be downloaded at: https://www.mdpi.com/article/10.3390/ijerph23040455/s1, Table S1: Data collection questionnaire; Table S2: Association between participant characteristics and 10-year risk for developing type 2 diabetes among Kuwait University employees; Table S3: Sensitivity analysis using multivariable logistic regression with a ≥9 FINDRISC threshold (*n* = 407); Dataset S1: De-identified cleaned dataset used in the analysis.

## Abbreviations

The following abbreviations are used in this manuscript:

T2DM	Type 2 Diabetes Mellitus
FINDRISC	Finnish Diabetes Risk Score
WHO	World Health Organization
IDF	International Diabetes Federation
KU	Kuwait University
MENA	Middle East and North Africa
BMI	Body Mass Index
HSC	Health Sciences Center
SD	Standard Deviation
aOR	Adjusted Odds Ratio
CI	Confidence Interval
VIF	Variance inflation
ROC	Receiver operating characteristic
PhD	Doctor of Philosophy
MD	Doctor of Medicine
DDS	Doctor of Dental Surgery
BP	Blood Pressure
GCC	Gulf Cooperation Council
