# Supplementary material for: Assessing the Risk of Type 2 Diabetes Among University Employees in Kuwait: A Cross-Sectional Study
Source: Int J Environ Res Public Health. 2026 Apr 2;23(4):455. doi: 10.3390/ijerph23040455 (PMC13115712; doi:10.3390/ijerph23040455)
Supplement: Supplementary file 1 [file ijerph-23-00455-s001.zip › Supplementary 1 Table S1 Data Collection Questionnaire.pdf]

Table S1: Data collection questionnaire

(English Version)
1. Have you answered this questionnaire before?<br>- Yes<br>- No
2. Have you been diagnosed with diabetes?<br>- Yes<br>- No
3. Are you currently an employee at Kuwait University?<br>- Yes<br>- No
4. What is your gender?<br>- Male<br>- Female
5. Are you currently pregnant? (for female participants only)<br>- Yes<br>- No
6. What is your nationality?<br>- Kuwaiti<br>- non-Kuwaiti
7. How old are you?<br>- 18-44 years<br>- 45-54 years<br>- 55-64 years<br>- 65 years and older
8. How much do you weigh? (in kilograms)<br>- (Participants insert their weight).
9. What is your height? (in centimeters)<br>- (Participants insert their height).

10. What is your waist circumference? *
    - Less than 80 cm (less than 31 inches)
    - 80-88 cm (31-35 inches)
    - 89-93 cm (35-36 inches)
    - 94-102 cm (37-40 inches)
    - More than 102 cm (more than 40 inches)

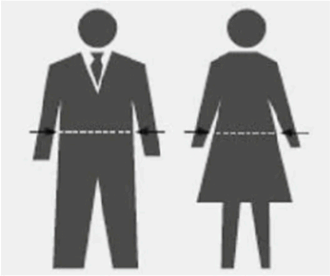

11. What is your marital status?
    - Single
    - Married
    - Divorced
    - Widowed

12. What is your professional position in Kuwait University?
    - Academic (e.g., professors, teaching assistants, lecturers, academic administrators, research faculty).
    - Non-academic (e.g., administrative staff, student affair staff, human resources staff, technical and IT staff, facilities management staff (security, maintenance)).

13. What is your highest level of education completed?
    - High school diploma or less
    - 2-Year college diploma
    - Bachelor's degree
    - Master's degree
    - Doctoral degree and/or professional degree (PhD, MD, DDS, etc.)

14. Are you physically active for at least 30 minutes every day?
    - Yes
    - No

15. How often do you eat fruits and vegetables?
    - Every day
    - Not every day

16. What is your current smoking status?
    - Smoker (i.e., smoked at least 100 cigarettes in your lifetime and currently smoke)
    - Former smoker (i.e., smoked at least 100 cigarettes in your lifetime but had quit smoking)
    - Never smoker (i.e., never smoked, or have smoked less than 100 cigarettes in your lifetime)

17. Have you ever taken medication for high blood pressure on a regular basis?
    - Yes
    - No

18. Have you ever been found to have high blood glucose (e.g., in a health examination, during an illness, during pregnancy)?
    - Yes
    - No

19. Have any of the members of your immediate family or other relatives been diagnosed with diabetes (type 1 or type 2)?
    - No
    - Yes, grandparent, aunt, uncle, or first cousin
    - Yes, parent, brother, sister, or own child

20. In your opinion, how likely are you to develop Type 2 Diabetes in the next 10 years, considering your family medical history and lifestyle factors?
    - Not at all likely
    - Somewhat likely
    - Very likely
    - I don't know

21. To what extent do you believe that the university supports its workers in overcoming barriers related to health and well-being?
    - Strongly agree
    - Agree
    - Neutral
    - Disagree
    - Strongly disagree

Note: FINDRISC scoring: Points were assigned according to the original Finnish Diabetes Risk Score algorithm:
- age (<45 = 0; 45–54 = 2; 55–64 = 3; ≥65 = 4);
- BMI (<25 = 0; 25–29.9 = 1; ≥30 = 3);
- waist circumference (sex-specific thresholds: men <94 = 0, 94–102 = 3, >102 = 4; women <80 = 0, 80–88 = 3, >88 = 4);

- ≥30 min daily physical activity (yes = 0, no = 2);
- daily fruit/vegetable consumption (yes = 0, no = 1);
- antihypertensive medication use (no = 0, yes = 2);
- history of elevated blood glucose (no = 0, yes = 5);
- family history of diabetes (none = 0, second-degree relative = 3, first-degree relative = 5).

Total scores range from 0–26, with higher scores indicating greater estimated 10-year risk of developing type 2 diabetes.

* Waist circumference was categorized according to the original FINDRISC sex-specific thresholds. For men, cutoffs were <94 cm (0 points), 94–102 cm (3 points), and >102 cm (4 points). For women, cutoffs were <80 cm (0 points), 80–88 cm (3 points), and >88 cm (4 points). Scoring was applied accordingly in the calculation of the total FINDRISC score.

(Arabic Version)

1. هل أجبت على هذا الاستبيان من قبل؟
- نعم
- لا

2. هل تم تشخيصك بمرض السكري؟
- نعم
- لا

3. هل أنت موظف في جامعة الكويت؟
- نعم
- لا

4. ما هو جنسك؟
- ذكر
- أنثى

5. هل أنتِ حامل؟ (هذا السؤال مخصص للمشاركات النساء فقط)
- نعم
- لا

6. ما هي جنسيتك؟
- كويتي
- غير كويتي

7. كم عمرك؟
- 18 - 44 سنة
- 45 - 54 سنة
- 55 - 64 سنة
- 65 عاما فما فوق

8. كم وزنك؟ (كيلوغرا□)
(ادخال الوزن)

9. ما هو طولك؟ (سنتيمتر)
(ادخال الطول)

10. ما هو قياس محيط الخصر؟ *
- أقل من 80 سم (31 إنش/ بوصة)
- 80 - 88 سم (31 - 35 إنش/ بوصة)
- 89 - 93 سم (35 - 36 إنش/ بوصة)
- 94 - 102 سم (37 - 40 إنش/ بوصة)
- أكثر من 102 سم (40 إنش/ بوصة)

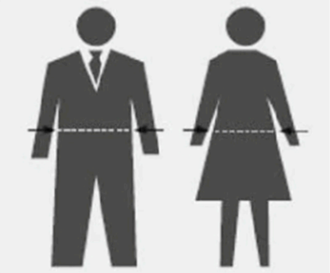

11. ما هي حالتك الاجتماعية؟
- أعزب
- متزوج
- مطلق
- أرمل

12. ما هو منصبك المهني في جامعة الكويت؟
- أكاديمي (مثل الأساتذة ومساعدي التدريس والمحاضرين والإداريين الأكاديميين وأعضاء هيئة التدريس).
- غير أكاديمي (مثل الموظفين الإداريين، وموظفي شؤون الطلاب، وموظفي الموارد البشرية، والموظفين التقنيين وموظفي تكنولوجيا المعلومات، وموظفي إدارة المرافق، والأمن، والصيانة).

13. ما هو أعلى مستوى تعليمي حصلت عليه؟
- دبلو□ المدرسة الثانوية أو أقل
- دبلو□ الكلية سنتين
- درجة البكالوريوس
- ماجستير
- درجة الدكتوراه أو درجة مهنية (على سبيل المثال PhD, MD, DDS)

14. هل تمارس عادة ما لا يقل عن ٣٠ دقيقة من النشاط البدني اليومي في العمل و/أو أثناء وقت الفراغ (بما في ذلك النشاط اليومي العادي)؟
- نعم
- لا

15. كم مرة تأكل الخضارأو الفاكهة أو الفريز أو التوت؟
- كل يو□
- ليس يومياً

16. هل أنت مدخن؟
- نعم، مدخن (أي دخنت مئة سيجارة على الأقل في حياتك ومازلت تدخن)
- مدخن سابق (أي دخنت مئة سيجارة على الأقل في حياتك ولكنك أقلعت عن التدخين)
- غير مدخن (أي لم تدخن قط أو دخنت أقل من مئة سيجارة في حياتك)

17. هل سبق لك أن تناولت دواء لارتفاع ضغط الد□ بشكل منتظم؟
- نعم
- لا

18. هل سبق أن عانيت من ارتفاع نسبة الجلوكوز (السكر) في الد[illegible] (على سبيل المثال: في الفحص الصحي، أو أثناء المرض، أو أثناء الحمل)؟
- نعم
- لا

19. هل تم تشخيصك أنت أو أي فرد آخر من أفراد عائلتك المباشرين أو أقاربك الآخرين بمرض السكري؟
- لا
- نعم: الجد، أو الجدة، أو العمة، أو العم، أو الخال، أو الخالة، أو أحد أبناء العم، أو العمة، أو أحد أبناء الخال، أو الخالة.
- نعم: الوالد، أو الوالدة، أو الأخ، أو الأخت، أو أحد أبنائك.

20. في رأيك: ما مدى احتمالية إصابتك بمرض السكري من النوع الثاني خلال السنوات العشر القادمة؟ مع الأخذ بالاعتبار تاريخ عائلتك الطبي وعوامل نمط حياتك؟
- غير محتمل على الإطلاق
- محتمل إلى حد ما
- محتمل جدا
- لا أعلم

21. إلى أي مدى تعتقد أن الجامعة تدعم العاملين بها في التغلب على العوائق المتعلقة بالصحة؟
- أوافق بشدة
- أوافق إلى حد ما
- محايد
- لا أوافق إلى حد ما
- لا أوافق بشدة

* تم تصنيف محيط الخصر وفقًا للقيم المعتمدة الأصلية الخاصة لكل من الرجال والنساء في مقياس FINDRISC بالنسبة للرجال، كانت نقاط القطع هي: أقل من 94 سم (0 نقاط)، من 94 إلى 102 سم (3 نقاط)، وأكثر من 102 سم (4 نقاط). أما بالنسبة للنساء، فكانت نقاط القطع هي: أقل من 80 سم (0 نقاط)، من 80 إلى 88 سم (3 نقاط)، وأكثر من 88 سم (4 نقاط). وتم تطبيق نظ[illegible] احتساب النقاط وفقًا لذلك عند حساب الدرجة الكلية لمقياس FINDRISC

تتراوح الدرجات الإجمالية من 0 إلى 26، وتشير الدرجات الأعلى إلى زيادة خطر الإصابة بداء السكري من النوع 2 خلال 10 سنوات.
