# Supplementary material for: Assessing the Risk of Type 2 Diabetes Among University Employees in Kuwait: A Cross-Sectional Study
Source: Int J Environ Res Public Health. 2026 Apr 2;23(4):455. doi: 10.3390/ijerph23040455 (PMC13115712; doi:10.3390/ijerph23040455)
Supplement: Supplementary file 1 [file ijerph-23-00455-s001.zip › Supplementary 2 Table S2 Association between participant characteristics and 10-year risk for developing Type 2 diabetes among Kuwait university employees (n=407) .pdf]

Table S2: Association between participant characteristics and 10-year risk for developing Type 2 diabetes among Kuwait University employees (*n*=407)

Characteristics	*n* (%)	10-year risk for developing Type 2 diabetes		P-value
		**not at increased risk (FINDSRISC ≤ 11) *n* (%) (*n* = 270)**	**at increased risk (FINDSRISC ≥ 12) *n* (%) (*n* = 137)**	
Gender				
Female	236 (58.0)	142 (52.6)	94 (68.6)	<0.001*
Male	171 (42.0)	128 (47.4)	43 (31.4)	
Age (years)				
18-44	264 (64.9)	197 (73.0)	67 (48.9)	<0.001*
45-54	89 (21.9)	44 (16.3)	45 (32.9)	
> 54 †	54 (13.3)	29 (10.7)	25 (18.3)	
Nationality				
Kuwaiti	274 (67.3)	181 (67.0)	93 (67.9)	0.863
Non-Kuwaiti	133 (32.7)	89 (33.0)	44 (32.1)	
Marital status				
Single	102 (25.1)	83 (30.7)	19 (13.9)	<0.001*
Married	279 (68.6)	170 (63.0)	109 (79.6)	
Divorced/widowed	26 (6.4)	17 (6.3)	9 (6.6)	
Professional position				
Academic	188 (46.2)	122 (45.2)	66 (48.2)	0.567
Non-academic	219 (53.8)	148 (54.8)	71 (51.8)	
Education level				
2-Year college diploma or less	58 (14.3)	44 (16.3)	14 (10.2)	0.232
Bachelor's degree	142 (34.9)	97 (35.9)	45 (32.9)	
Master's degree	68 (16.7)	41 (15.2)	27 (19.7)	
Doctoral degree and/or professional degree (PhD, MD, DDS, etc.)	139 (34.2)	88 (32.6)	51 (37.2)	
Smoking status				
Smoker	38 (9.4)	26 (9.6)	12 (8.8)	0.845
Former smoker	24 (5.9)	17 (6.3)	7 (5.1)	
Never smoked	345 (84.8)	227 (84.1)	118 (86.1)	
Employee perception of diabetes risk				
Not at all likely	116 (28.5)	90 (33.3)	26 (19.0)	<0.001*
Somewhat likely	169 (41.5)	107 (39.6)	62 (45.3)	
Very likely	42 (10.3)	15 (5.6)	27 (19.7)	

I don’t know	80 (19.7)	58 (21.5)	22 (16.1)	
Employee perception of workplace health and wellbeing support				
Strongly agree	54 (13.3)	37 (13.7)	17 (12.4)	0.137
Agree	99 (24.3)	63 (23.3)	36 (26.3)	
Neutral	131 (32.2)	96 (35.6)	35 (26.3)	
Disagree	52 (12.8)	28 (10.4)	24 (17.5)	
Strongly disagree	71 (17.4)	46 (17.0)	25 (18.3)	

n: Number of participants; %: Overall percentage; FINDRISC: Finnish Diabetes Risk Score; PhD: Doctor of Philosophy; MD: Doctor of Medicine; DDS: Doctor of Dental Surgery; *: Statistically significant at $p < 0.05$; †: Seven participants were aged >64 years were grouped with the >54 years category but they were assigned a FINDRISC age score of 4 points for statistical analysis.
