# Supplementary material for: Assessing the Risk of Type 2 Diabetes Among University Employees in Kuwait: A Cross-Sectional Study
Source: Int J Environ Res Public Health. 2026 Apr 2;23(4):455. doi: 10.3390/ijerph23040455 (PMC13115712; doi:10.3390/ijerph23040455)
Supplement: Supplementary file 1 [file ijerph-23-00455-s001.zip › Supplementary 3 Table S3 Sensitivity analysis using multivariable logistic regression with a ≥9 FINDRISC threshold (n=407)..pdf]

Table S3: Sensitivity analysis using multivariable logistic regression with a ≥9 FINDRISC threshold (n=407).

Variable	Adjusted Odds Ratio	95% CI	P-value
Gender			
Male	Reference		
Female	3.83	2.14-6.83	<0.001*
Nationality			
Kuwaiti	Reference		
Non-Kuwaiti	1.89	1.09-3.27	0.023*
Marital status			
Single	Reference		
Married	2.16	1.25-3.72	0.006*
Divorced/widowed	1.95	0.70-5.40	0.202
Professional position			
Academic	Reference		
Non-academic	0.92	0.43-1.96	0.825
Education level			
2-Year college diploma or less	Reference		
Bachelor's degree	1.20	0.58-2.47	0.621
Master's degree	1.20	0.48-2.99	0.692
Doctoral degree and/or professional degree (PhD, MD, DDS, etc.)	1.91	0.70-5.26	0.205
Smoking status			
Never smoked	Reference		
Smoker/former smoker	0.94	0.48-1.84	0.856
Employee perception of diabetes risk			
Not at all likely	Reference		
Somewhat likely	3.61	2.09-6.26	< 0.001*
Very likely	12.82	4.58-35.85	< 0.001*
I don't know	2.08	1.09-3.96	0.026*
Employee perception of workplace health and wellbeing support			
Strongly agree	Reference		
Agree	1.27	0.58-2.78	0.554
Neutral	0.86	0.41-1.83	0.700
Disagree	1.02	0.42-2.45	0.968
Strongly disagree	1.96	0.83-4.60	0.123

CI: Confidence interval; PhD: Doctor of Philosophy; MD: Doctor of Medicine; DDS: Doctor of Dental Surgery; *: Statistically significant at $p < 0.05$; T2DM: Type 2 Diabetes Mellitus; FINDRISC: Finnish Diabetes Risk Score.
